# A segmental duplication encompassing *S*-haplotype triggers pollen-part self-compatibility in Japanese pear (*Pyrus pyrifolia*)

**DOI:** 10.1007/s11032-013-9938-5

**Published:** 2013-08-21

**Authors:** Nobuko Mase, Yutaka Sawamura, Toshiya Yamamoto, Norio Takada, Sogo Nishio, Toshihiro Saito, Hiroyuki Iketani

**Affiliations:** 1 NARO Institute of Fruit Tree Science, National Agriculture and Food Research Organization, 2-1 Fujimoto, Tsukuba, Ibaraki 305-8605 Japan; 2 Graduate School of Life and Environmental Science, University of Tsukuba, Tennoudai 1-1-1, Tsukuba, Ibaraki 305-8572 Japan

**Keywords:** Competitive interaction, γ-Irradiation, Pollen-part self-compatible mutant, *Pyrus pyrifolia*, Segmental duplication, Self-incompatibility

## Abstract

Self-compatible mutants of self-incompatible crops have been extensively studied for research and agricultural purposes. Until now, the only known pollen-part self-compatible mutants in Rosaceae subtribe Pyrinae, which contains many important fruit trees, were polyploid. This study revealed that the pollen-part self-compatibility of breeding selection 415-1, a recently discovered mutant of Japanese pear (*Pyrus pyrifolia*) derived from γ-irradiated pollen, is caused by a duplication of an *S*-haplotype. In the progeny of 415-1, some plants had three *S*-haplotypes, two of which were from the pollen parent. Thus, 415-1 was able to produce pollen with two *S*-haplotypes, even though it was found to be diploid: the relative nuclear DNA content measured by flow cytometry showed no significant difference from that of a diploid cultivar. Inheritance patterns of simple sequence repeat (SSR) alleles in the same linkage group as the *S*-locus (LG 17) showed that some SSRs closely linked to *S*-haplotypes were duplicated in progeny containing the duplicated *S*-haplotype. These results indicate that the pollen-part self-compatibility of 415-1 is not caused by a mutation of pollen *S* factors in either one of the *S*-haplotypes, but by a segmental duplication encompassing the *S*-haplotype. Consequently, 415-1 can produce *S*-heteroallelic pollen grains that are capable of breaking down self-incompatibility (SI) by competitive interaction between the two different *S* factors in the pollen grain. 415-1 is the first diploid pollen-part self-compatible mutant with a duplicated *S*-haplotype to be discovered in the Pyrinae. The fact that 415-1 is not polyploid makes it particularly valuable for further studies of SI mechanisms.

## Introduction

Self-incompatibility (SI) is a reproductive strategy to prevent inbreeding and maintain genetic diversity in flowering plants (de Nettancourt 2001). The families Solanaceae, Rosaceae, and Plantaginaceae exhibit *S*-RNase-based SI that is gametophytically controlled by a multigene complex, the *S*-locus. The genes at this locus encode *S*-RNase (McClure et al. 1989; Sassa et al. 1996; Xue et al. 1996) as a stylar factor and F-box proteins called *S*-locus F-box (SLF) in an *Antirrhinum* interspecific hybrid (Lai et al. 2002), *Prunus mume* Siebold & Zucc. (Entani et al. 2003), and *Petunia inflata* R.E. Fr. (Sijacic et al. 2004) or SFB (*S* haplotype-specific F-box protein) in *Prunus dulcis* D.A. Webb (Ushijima et al. 2003) as pollen factors for self/non-self recognition. These two genes at the *S*-locus segregate as a single unit referred to as the *S*-haplotype. When a pollen *S*-allele matches either one of the *S*-alleles of the pistil, *S*-RNase secreted by the pistil tissue degrades the ribosomal RNA in the pollen tube produced by that pollen grain, resulting in the inhibition of pollen tube elongation and the prevention of fertilization.

Although advantageous from the standpoint of genetic diversity, SI is disadvantageous for agriculture. Thus, self-compatible mutants have been explored for use in agricultural production, particularly in self-incompatible commercial fruit tree species. Several spontaneous self-compatible mutants have been used for agricultural production and breeding material, and as experimental material for studying the genes controlling SI (Norioka et al. 1996; Yamane et al. 2003). In addition, much effort has been devoted to obtaining self-compatible mutants by using chemical mutagens or ionizing radiation (Ushijima et al. 2004; Sonneveld et al. 2005).

Self-compatible mutants in *S*-Rnase-based self-incompatible plants are classified as either stylar-part or pollen-part. Stylar-part self-compatible mutants are caused by deletion or non-functional mutation of an *S*-*RNase* gene (Royo et al. 1994; Sassa et al. 1997). The causes of pollen-part self-compatible mutants (PPMs) are not well understood. At present, PPMs are classified broadly into two categories based on the mechanism by which incompatibility is lost. In the first category, breakdown of pollen *S* function is thought to occur because of competition between two different pollen *S* factors (assumed to be F-box proteins) within individual pollen grains by polyploidization or segmental duplication of the chromosome containing the *S*-locus. In the second category, loss of pollen *S* function is caused by genetic deletion or transposon insertion into *S*-related F-box genes.

In the Solanaceae and in subtribe Pyrinae (formerly Maloideae) of the Rosaceae, polyploid mutants with more than two *S*-alleles exhibit pollen-part SC (Lewis and Modlibowska 1942; de Nettancourt et al. 1971; Adachi et al. 2009). Furthermore, diploid mutants with a duplicated *S*-allele obtained in the progenies of irradiated *S*-heterozygous diploids also become pollen-part self-compatible (Brewbaker and Natarajan 1960; Pandey 1965). This phenomenon was termed “competition” or “competitive interaction (CI)” by Lewis and Modlibowska (1942) and Lewis (1952) because they hypothesized that both pollen *S* functions were broken down when two different alleles existed in a single pollen grain. In the species that have PPM caused by a duplicated *S*-haplotype, the pollen *S*-related F-box genes are called *S*-haplotype-specific F-box brothers (*SFBB*), *SLF*, or *SLF*-like genes. These genes exhibit specific expression in pollen and have allele-specific diversity among *S*-haplotypes (Sassa et al. 2007; Minamikawa et al. 2010; Kubo et al. 2010; de Franceschi et al. 2011).

Recently, Kubo et al. (2010) constructed petunia transformants that produced pollen with a transgenic copy of *S*_*7*_-*SLF1* (one of the *SLF*-like genes of the *S*_*7*_-haplotype) and reported that pollen with an *S*_*9*_-haplotype (and also containing the *S*_*7*_-*SLF1* transgene) induced SC whereas pollen with an *S*_*5*_-, *S*_*11*_-, or *S*_*19*_-haplotype (and the *S*_*7*_-*SLF1* transgene) did not. Furthermore, Kakui et al. (2011) and Saito et al. (2012) reported that the pollen of a stylar-part self-compatible mutant cultivar of Japanese pear (*Pyrus pyrifolia* Nakai) that lacks both *S*_*4*_-*RNase* and *PpSFBB* ^4-d1^ (one of the *P. pyrifolia* *SFBB* genes) (Okada et al. 2008, 2011) and contains a mutated *S*_*4*_ (*S*_*4*_^*sm*^) haplotype showed cross-incompatibility with a style harboring a non-*S*_*4*_ (e.g., *S*_*1*_) haplotype.

From these results, Kubo et al. (2010) hypothesized that in species within the Solanaceae and Pyrinae, each of the *S*-related F-box proteins (SLFs/SFBBs) can degrade only some of the non-self *S*-RNases, but that the complete set of SLFs/SFBBs in the *S*-haplotypes of a given species is capable of degrading all of the non-self *S*-RNases in that species. If this hypothesis is correct, PPMs with a duplicated *S*-haplotype could produce pollen grains harboring two different *S*-haplotypes that together can degrade all *S*-RNases, including their own, and become self-compatible.

On the other hand, there are some reports in *Prunus* that pollen with two different *S*-haplotypes does not show CI (Hauck et al. 2006; Bošković et al. 2006). Instead, these PPMs have mutations in one of the F-box protein genes. Thus, in these instances the F-box protein (SLF/SFB) is considered to be the sole pollen *S* factor, and a specific *S*-haplotype can become self-compatible by silencing or dysfunction of the pollen *S* factor (Yamane et al. 2003; Ushijima et al. 2004).

These different mechanisms by which SC can arise from SI in different species raise the possibility of different patterns of self/non-self recognition and mechanisms of inactivation of *S*-RNase in self-incompatible responses. Various PPMs from different self-incompatible species are necessary to elucidate the mechanism of SI by studying the mutations in the genes involved in SC and their original functions in the self-incompatible progenitor.

Until now, all of the known PPMs in the Pyrinae have been tetraploids (Crane and Lewis 1942; Adachi et al. 2009; Tahira et al. 2010). Because of their ploidy, these materials are unsuitable for use in research to identify the gene responsible for pollen-part SI, and they are difficult to use in breeding to generate self-compatible cultivars by crossing with normal diploids. On the other hand, diploid PPMs with mutations in the gene for pollen recognition or with segmental duplications involving the *S*-locus could contribute to the identification of genomic sequences and gene functions that control pollen-part SC as well as providing suitable material for cultivar improvement.

A self-compatible mutant selection of Japanese pear (designated 415-1) with apparent *S*_*4*_- and *S*_*5*_-*RNase* genotypes was identified in the progeny of a cross using pollen from a tree continuously exposed to low-dose-rate γ-irradiation. Reciprocal crosses with self-incompatible cultivars with the same *S*-alleles indicated that this mutant is a PPM that lost its pollen SI function but retained its stylar SI function (Sawamura et al. 2013). However, since the pollen of a PPM is cross-compatible with the styles of all *S*-*RNase* genotypes, the identity of the pollen *S*-allele mutated in 415-1 could not be determined by cross-compatibility tests.

The purpose of the present study was to elucidate the cause for SC in 415-1 by determining whether it was caused by a loss of function of a pollen *S* factor or by CI in heteroallelic diploid pollen (produced by polyploidization or segmental duplication involving the *S*-locus). The following analyses were performed: (1) DNA ploidy analysis, (2) segregation analysis of *S*-haplotypes, and (3) segregation analysis of genetic markers around the *S*-locus.

## Materials and methods

### Preparation of self and outcross progeny

The PPM selection 415-1 was generated as a result of selective fertilization with self-compatible pollen from γ-irradiated cultivar Kosui (*S*_*4*_ *S*_*5*_) (SI) by pollinating the style of non-irradiated Kosui (Sawamura et al. 2013). Three populations were generated from crosses using 415-1 as the pollen parent in order to identify the pollen-part mutated allele (Table 1).

**Table 1:** Discrepancy between observed segregation of the electrophoretic *S*-phenotype and the expected *S*-haplotype under the hypothesis of pollen *S*-factor mutation

Parents and *S*-haplotypes	Number of seedlings	Hypothesized 415-1 *S*-haplotype^a^	Expected segregation of *S*-haplotype	Observed segregation of electrophoretic *S*-phenotype	Goodness of fit (*p*)^b^
415-1 (*S*_*4*_ *S*_*5*_) PPM × self	23	*S*_*4*_^*pm*^ *S*_*5*_	*S*_*4*_^*pm*^ *S*_*4*_^*pm*^:*S*_*4*_^*pm*^ *S*_*5*_ = 1:1	*S*_*4*_ *S*_*5*_ = 23	**
		*S*_*4*_ *S*_*5*_^*pm*^	*S*_*4*_ *S*_*5*_^*pm*^:*S*_*5*_^*pm*^ *S*_*5*_^*pm*^ = 1:1		**
Syuugyoku (*S*_*4*_ *S*_*5*_) SI × 415-1 (*S*_*4*_ *S*_*5*_) PPM	63	*S*_*4*_^*pm*^ *S*_*5*_	*S*_*4*_ *S*_*4*_^*pm*^:*S*_*4*_^*pm*^ *S*_*5*_ = 1:1	*S*_*4*_ *S*_*5*_:*S*_*5*_ *S*_*5*_ = 60:3	**
		*S*_*4*_ *S*_*5*_^*pm*^	*S*_*4*_ *S*_*5*_^*pm*^:*S*_*5*_ *S*_*5*_^*pm*^ = 1:1		**
Niitaka (*S*_*3*_ *S*_*9*_) SI × 415-1 (*S*_*4*_ *S*_*5*_) PPM	103	*S*_*4*_^*pm*^ *S*_*5*_	*S*_*3*_ *S*_*4*_^*pm*^:*S*_*3*_ *S*_*5*_:*S*_*4*_^*pm*^ *S*_*9*_:*S*_*5*_ *S*_*9*_ = 1:1:1:1	*S*_*3*_ *S*_*4*_:*S*_*3*_ *S*_5_:*S*_*4*_ *S*_*9*_:*S*_*5*_ *S*_*9*_:*S*_*3*_ *S*_*4*_ *S*_*5*_:*S*_*4*_ *S*_*5*_ *S*_*9*_ = 2:40:0:47:7:7	**^c^
		*S*_*4*_ *S*_*5*_^*pm*^	*S*_*3*_ *S*_*4*_:*S*_*3*_ *S*_*5*_^*pm*^:*S*_*4*_ *S*_*9*_:*S*_*5*_^*pm*^ *S*_*9*_ = 1:1:1:1		**^c^

** Significantly different from the expected segregation ratio (*p* < 0.01)

^a^ *S*_*4*_^*pm*^ hypothesized pollen-part mutant of *S*_*4*_, *S*_*5*_^*pm*^ hypothesized pollen-part mutant of *S*_*5*_

^b^ *p* value for the binomial goodness-of-fit tests

^c^Multinomial goodness-of-fit tests were performed excluding the unexpected classes (triallelic electrophoretic *S*-phenotypes)

If 415-1 is self-compatible because of a mutation in one of the putative pollen factors of either the *S*_*4*_ or *S*_*5*_ haplotype, only pollen grains having a self-compatible allele would contribute to fertilization in a cross with a cultivar containing the same haplotypes, and the apparent *S*-haplotypes of the progeny (estimated from the genotype of markers representative of each *S*-haplotype) would segregate 1:1 for “homozygous” individuals (actually containing one mutant allele and one wild-type allele of the same *S*-haplotype) and heterozygous individuals (containing one mutant and one wild-type allele of different *S*-haplotypes).

In contrast, if 415-1 is self-compatible because of duplication of the *S*-haplotype, only the *S*_*4*_ *S*_*5*_ heteroallelic pollen grains would contribute to fertilization of a cultivar containing the same (*S*_*4*_ and *S*_*5*_) haplotypes, and all of the progeny would be *S*_*4*_ *S*_*5*_ heterozygous. In addition, some plants with three *S*-haplotypes would be obtained in the progeny of crosses to cultivars that share no *S*-haplotype with 415-1 (i.e., that contain neither the *S*_*4*_ nor the *S*_*5*_ haplotype).

Therefore, two populations, (1) 23 self-progeny of 415-1 and (2) 63 F1 plants obtained from a cross of 415-1 to the self-incompatible cultivar Syuugyoku (*S*_*4*_ *S*_*5*_) were used to determine the type of self-compatible allele present in 415-1. In addition, 103 F1 plants obtained from a cross to the self-incompatible cultivar Niitaka (*S*_*3*_ *S*_*9*_), which does not have any *S*-haplotypes in common with 415-1, were used to further examine the inheritance of the self-compatible allele (Table 1).

### DNA extraction

Genomic DNA was extracted using a FastDNA kit (MP Biomedicals, USA) according to the manufacturer’s instructions, except that 10 mg polyvinylpyrrolidone (insoluble) and 30 μL 2-mercaptoethanol were added to Cell Lysis Solution (0.8 mL CLS-VF, 0.2 mL PPS) in the initial homogenization step.

### Determination of the *S*-haplotypes of progenies

CAPS (cleaved amplified polymorphic sequence) analysis was used to determine the *S*-*RNase* and *PpSFBB* ^−*γ*^ (a F-box protein gene) alleles present in the *S*-haplotypes of the parents and progenies described above. The results of this analysis are referred to here as “electrophoretic *S*-phenotypes”.

CAPS analysis of *S*-*RNase* followed the procedure of Takasaki et al. (2004). Partial sequences of the *S*-*RNase* gene were amplified by PCR using FTQQYQ and anti-I(T)IWPNV primers. *S*-*RNase* allele-specific fragments were detected by digestion with *Nde*I for *S*_*4*_, *Alw*NI for *S*_*5*_, and a combination of *Nde*I and *Alw*NI for *S*_*3*_ and *S*_*9*_.

CAPS analysis of *PpSFBB* ^−*γ*^ followed the procedure of Kakui et al. (2007). Partial sequences of *PpSFBB* ^−*γ*^ were amplified by PCR using the primers PpFBXf7 and PpFBXr3. *PpSFBB* ^−*γ*^ allele-specific fragments were detected by digestion with *Nsp*I for *S*_*4*_, *Afl*II for *S*_*5*_, *Hae*III for *S*_*9*_, and *Hpy*CH4IV for *S*_*3*_ (Hiroyuki Kakui [Nagoya University], personal communication).

PCR amplification was performed in a total volume of 20 μL of 1× *Ex Taq* buffer (TaKaRa Bio, Japan) containing 15 ng of genomic DNA, 1 U of TaKaRa *Ex Taq* polymerase (TaKaRa Bio), 0.2 mM of each dNTP, and 0.5 μM of each forward primer and reverse primer. PCR was carried out for 30 cycles of denaturation at 94 °C for 15 s, annealing at 54 °C for 15 s for *S*-*RNase* or 59 °C for 15 s for *PpSFBB* ^−*γ*^, and extension at 72 °C for 1.5 min, followed by a final extension at 72 °C for 7 min. The PCR products were incubated with the specified endonucleases for 16 h at 37 °C. These products were separated on 2 % agarose gels in TBE buffer and visualized with ethidium bromide. The goodness of fit of the progeny segregation ratios to the expected Mendelian ratios was tested using binomial or multinomial exact tests.

### Estimation of ploidy level

Flow cytometry was performed to measure the relative nuclear DNA content of 415-1 and Kosui (diploid) using maize (*Zea mays* L.) inbred line B73, which has an actual nuclear DNA content of 2.3 Gbp (Schnable et al. 2009), as an internal standard. Cuttings of pear and seedlings of maize were grown in a growth chamber (20 °C, 12-h light period). About 0.15 cm^2^ of pear leaf and 1.5 cm^2^ of maize leaf were placed together in 400 μL of extraction buffer from the Partec CyStain UV Precise P kit (Partec GmbH, Germany) with 1 % polyvinylpyrrolidone K-30 (Wako Pure Chemical Industries Ltd., Japan) and chopped using a razor blade. The suspension was incubated for 1 min at 4 °C and then filtered through a 30-μm nylon mesh (Partec Gmbh). Then, 1.6 mL of staining buffer containing 4,6-diamidino-2-phenylindole (DAPI) was added and the mixture was incubated for 5 min at 4 °C in the dark. Data were collected for approximately 5,000 nuclei per sample using a flow cytometer (Ploidy Analyser PA-II, Partec GmbH) with UV excitation at 366 nm from a mercury arc lamp. The histograms were generated on a linear scale, and 15 measurements with coefficients of variation (CVs) smaller than 7 % were obtained by using five different leaves from each pear cultivar on three different days. Comparisons of ploidy levels between the two pear cultivars were expressed in arbitrary units (AU) that represent the ratio (%) of the mode value of fluorescence intensity of the *G*_0_/*G*_1_ peak of pear to that of the internal standard (maize) in each measurement.

### SSR genotyping

Twenty-seven simple sequence repeat (SSR) markers, previously developed and mapped to the same linkage group as the *S*-haplotype [linkage group (LG) 17] of pear or apple (*Malus* × *domestica* Borkh.), were tested for the analysis of 415-1. These SSR markers consisted of 15 pear SSRs [HGT6, NH008b, NH014a, NH015a, NB125a, TsuENH002, TsuENH026, TsuENH028, TsuENH033, TsuENH071, and TsuENH080 (Yamamoto et al. 2002a, b, 2007; Nishitani et al. 2009)], and TsuENH104, TsuENH114, TsuENH154, and TsuENH163 [GenBank accession numbers AB735182, AB735183, AB735184, and AB735185, respectively]) and 12 apple SSRs [CH01b12, CH01h01, CH05g03, CH04c10, CN444542SSR, AF527800SSR, AT000174SSR, AJ001681SSR, AY187627SSR, NZmsMDAJ1681, NZmsEB137525, and NZmsEE663955 (Gianfranceschi et al. 1998; Liebhard et al. 2002; Silfverberg-Dilworth et al. 2006; Celton et al. 2009)].

PCR amplification was performed in 10 μL *Ex Taq* PCR buffer containing 0.2 mM of each dNTP, 0.5 μM of each forward primer labeled with a fluorescent chemical (FAM, TET, HEX, or VIC) and unlabeled reverse primer, 5 ng of genomic DNA, and 0.25 U of *Ex Taq* polymerase. PCR was carried out for 30 cycles of denaturation at 94 °C for 15 s, annealing at 55 °C for 15 s, and extension at 72 °C for 1.5 min, followed by a final extension at 72 °C for 7 min. The PCR products were separated and detected using an ABI PRISM 3100 Genetic Analyzer (Applied Biosystems, USA). The sizes of the amplified bands were determined based on an internal DNA size standard (GeneScan 400 HD ROX Size Standard [Applied Biosystems]) with GeneScan Software (Applied Biosystems). Allele size data (in bp) were rounded down to the nearest whole number. The frequencies of recombination between the *S*-*RNase* gene and SSR markers were translated into genetic distances using the Kosambi map function (Kosambi 1944), and the linkage phase of markers within LG 17 was determined.

## Results

### Segregation of *S*-*RNase* and *PpSFBB*^−*γ*^ in the progenies of crosses involving 415-1

All 23 self-progeny of 415-1 were *S*_*4*_ *S*_*5*_ heterozygous for both *S*-*RNase* and *PpSFBB* ^−*γ*^. Out of 63 F1 progeny of Syuugyoku (*S*_*4*_ *S*_*5*_) × 415-1, 60 were *S*_*4*_ *S*_*5*_ and three were homozygous for *S*_*5*_. In short, almost all progenies from either self-pollination of 415-1 or cross-pollination with a cultivar with the same *S*-haplotypes (*S*_*4*_ *S*_*5*_) were heterozygous. These segregation patterns did not fit the hypothesis that the PPM in 415-1 was caused by a loss of function of pollen *S* factor *S*_*4*_ or *S*_*5*_ (Table 1).

**Fig. 1:**
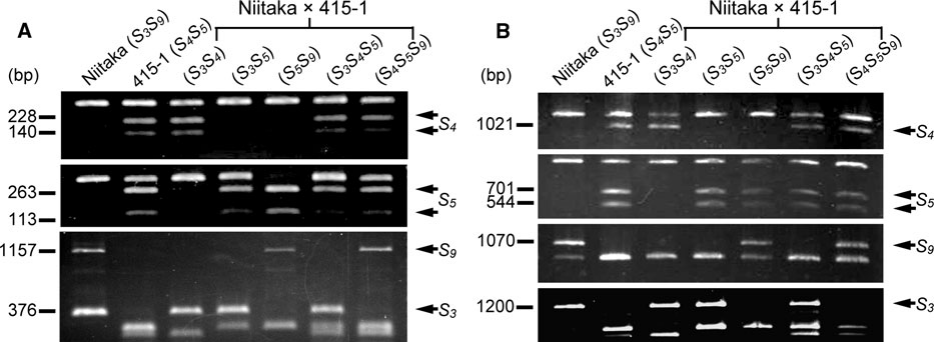
Segregation analysis of *S*-haplotypes by means of CAPS analysis of *S*-*RNase* (**a**) and *PpSFBB* ^−γ^ (**b**) alleles in the parents and five progeny plants of Niitaka × 415-1. The same plants are represented in both gels

In 103 F1 progeny obtained from a cross with the self-incompatible cultivar Niitaka (*S*_*3*_ *S*_*9*_), there was no recombination between the two CAPS markers (*S*-*RNase* and *PpSFBB* ^−*γ*^) in the *S*-haplotype. The electrophoretic *S*-phenotypes segregated in a ratio of 2 *S*_*3*_ *S*_*4*_:40 *S*_*3*_ *S*_*5*_:0 *S*_*4*_ *S*_*9*_:47 *S*_*5*_ *S*_*9*_:7 *S*_*3*_ *S*_*4*_ *S*_*5*_:7 *S*_*4*_ *S*_*5*_ *S*_*9*_ (Table 1; Fig. 1). Thus, 14 of the plants were triallelic for the *S*-haplotype.

### Ploidy level

Simultaneous analysis of pear and maize (internal standard) DAPI-stained nuclei in suspension produced histograms of fluorescence intensity with two peaks corresponding to the relative DNA content of the *G*_0_/*G*_1_ nuclei of both plants. The mean AU of 415-1 was 34.97 ± 1.36 % (CV: 5.80 ± 0.91 %), and the mean AU of diploid cultivar Kosui was 34.51 ± 1.81 % (CV: 5.55 ± 0.75 %). There was no significant difference in the ploidy status between 415-1 and Kosui (Student’s *t* test, Table 2).

**Table 2:** Relative nuclear DNA contents of 415-1 and Kosui determined by flow cytometric analysis

Cultivar	*n* ^a^	AU (%)^b^		CV (%)	Ploidy level
		Mean	SD		
415-1	15	35.1	1.19	5.80	2*n*
Kosui	15	34.8	1.36	5.55	2*n*

*CV* coefficient of variation of peak intensity

^a^ *n* number of samples (each from an individual leaf)

^*b*^ *AU* arbitrary unit, calculated as (peak value of relative fluorescence intensity of Japanese pear/peak value of relative fluorescence intensity of the internal standard, *Z. mays* B73) × 100

### Segregation of SSR markers on linkage group 17

**Fig. 2:**
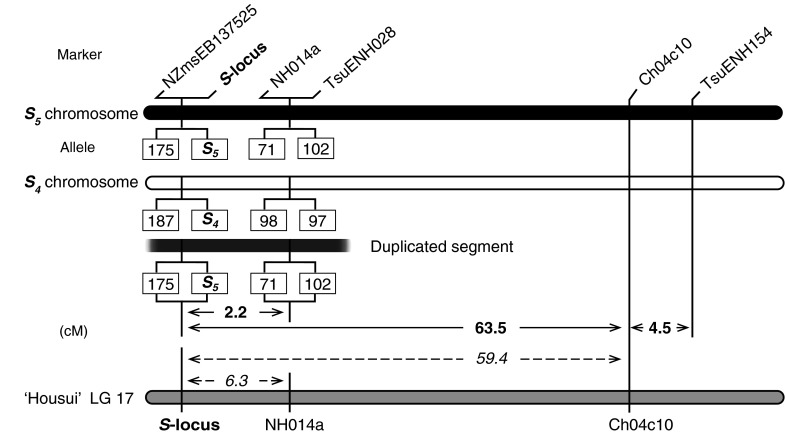
Local haplotype maps of 415-1 estimated from intermarker distances in the progeny of the Niitaka × 415-1 cross. Marker haplotypes are displayed as a pair of homologous LG 17 chromosomes and a duplicated segment of LG 17. Marker/allele designations connected by *horizontal lines* indicate that no recombination was observed between those markers. SSR alleles are designated by size (bp). On the basis of the genetic data, the duplicated segment is estimated to be contained within the chromosome containing *S*_*4*_. Only map distances are displayed for Ch04c10 and TsnENH154 because their linkage phases could not be inferred from the data. *Solid*-*line arrows* indicate the genetic distances calculated for the 89 biallelic progenies of the Niitaka × 415-1 cross. *Dashed*-*line arrows* indicate genetic distances from a high-density genetic map of the normal (self-incompatible) cultivar Housui (Terakami et al. 2009)

Of the 27 SSR markers we tested in LG 17, five (TsuENH154, CH04c10, NH014a, TsuENH028, and NZmsEB137525) showed heterozygosity in 415-1. These five markers were used to genotype the progeny from the cross of Niitaka (*S*_*3*_ *S*_*9*_) × 415-1. From this analysis, linkage maps of the *S*_*4*_ and *S*_*5*_ haplotypes were constructed (Fig. 2).

All 14 plants that were triallelic for the *S*-haplotype contained two alleles of NZmsEB137525 from the pollen parent 415-1 and one allele from the seed parent. For SSR markers NH014a and TsuENH028, 13 of the 14 plants contained two alleles from 415-1 and one contained only one allele from 415-1. On the other hand, at loci TsuENH154 and CH04c10, all 14 plants had just two alleles, one from the pollen parent and the other from the seed parent.

Two-point genetic linkage analysis was performed with the *S*-*RNase* gene and the five informative SSR markers by using the 89 progeny from the cross of Niitaka (*S*_*3*_ *S*_*9*_) × 415-1 that each had only two *S*-alleles, one from each parent. With this information, local haplotype maps of the region around the *S*-locus of 415-1 were constructed (Fig. 2). In 415-1, SSR markers NZmsEB137525, NH014a, and TsuENH028 were completely linked to the *S*-locus (i.e., no recombination was observed between the *S*-haplotype from 415-1 and these markers). In contrast, CH04c10 and TsuENH154 were located 63.5 and 67.9 cM, respectively, from the *S*-locus. The genetic distance between CH04c10 and TsuENH154 was 4.5 cM. In Niitaka, NZmsEB137525 was completely linked to the *S*-locus. However, segregation between the *S*-locus and markers NH014a and TsuENH028 was observed; these markers were estimated to be 2.2 cM from the *S*-locus. Genetic distances between the other two markers (CH04c10 and TsuENH154) and the *S*-locus could not be calculated for haplotypes from Niitaka because their genotypes were homozygous.

## Discussion

### Pollen-part self-compatibility of 415-1 is caused by duplication of an *S*-haplotype

In this study, the reason for the SC of 415-1 was determined by analyzing the segregation of *S*-haplotypes in the progeny and comparing the segregation ratios with the hypothesis of pollen *S*_*4*_ or *S*_*5*_ mutation. All 23 progeny from self-pollination of 415-1 (*S*_*4*_ *S*_*5*_) and 60 out of 63 progeny from cross-pollination to the cultivar Syuugyoku (also *S*_*4*_ *S*_*5*_) were heterozygous *S*_*4*_ *S*_*5*_ (Table 1); only three plants (from the cross) were homozygous (*S*_*5*_ *S*_*5*_). These relatively rare homozygous plants are assumed to be from *S*_*5*_ pollen that escaped degradation, as was previously reported in PPMs of *Nicotiana alata* Link and Otto (Pandey 1967), rather than from self-compatible pollen containing a mutated *S*_*5*_-factor. Instead, the observed segregation patterns support the hypothesis that PPM of 415-1 was caused by the duplication of an *S*-haplotype.

The inheritance of *S*-locus alleles from both parents was determined (Table 1) by analyzing the progeny obtained from a cross with the self-incompatible cultivar Niitaka (*S*_*3*_ *S*_*9*_), which does not share any *S*-haplotypes with 415-1. In the progeny, some plants contained three *S*-alleles: one allele (either *S*_*3*_ or *S*_*9*_) from the seed parent and both of the alleles (*S*_*4*_ *S*_*5*_) from the pollen parent. Therefore, 415-1 had the ability to produce *S*_*4*_ *S*_*5*_ pollen as well as *S*_*4*_ and *S*_*5*_ pollen. When 415-1 was used to pollinate a cultivar having the same *S*-haplotype (*S*_*4*_ *S*_*5*_), only the *S*_*4*_ *S*_*5*_ heteroallelic pollen would have been able to complete fertilization; thus, the *S*_*4*_ *S*_*5*_ heteroallelic progeny produced in such crosses are assumed to have been the product of an *S*_*4*_ or *S*_*5*_ egg cell and *S*_*4*_ *S*_*5*_ pollen. Such progeny would in fact have three *S*-alleles and their true genotypes would be *S*_*4*_ *S*_*4*_ *S*_*5*_ or *S*_*4*_ *S*_*5*_ *S*_*5*_.

In summary, the pollen-part SC of 415-1 was not caused by a mutation of a pollen *S* factor in either *S*-haplotype, but instead because a duplicated *S*-haplotype made it possible for 415-1 to produce *S*-heteroallelic pollen that expresses CI.

### Segmental duplication of an LG 17 region containing the *S*-haplotype

415-1 was estimated to be diploid because it showed no significant difference in relative nuclear DNA content from the diploid cultivar Kosui (Table 2). As a diploid, 415-1 would be expected to produce haploid pollen and diploid progeny. Nevertheless, two *S*-alleles from 415-1 were detected in some of its progeny (Table 1). This indicates that at least part of the chromosome corresponding to LG 17, including the *S*-haplotype, was duplicated, though the duplication was undetectable by flow cytometric analysis.

The haplotype linkage maps of LG 17 in 415-1 were very similar to that of the normal self-incompatible cultivar Housui (Terakami et al. 2009). Therefore, the chromosome corresponding to LG 17 of 415-1 is considered to be similar in overall structure to that of normal cultivars.

Three of the five informative SSR markers in LG 17 showed complete or very close linkage to the *S*-locus in 415-1. For these three markers, both of the alleles from 415-1 were detected in the progeny with the duplicated *S*-haplotype. This indicates that the duplicated part of the chromosome includes at least the *S*-locus and these three markers. In a previous molecular genetic analysis of *S*-haplotypes of Japanese pear (Kakui et al. 2011), *SFBB* genes were mapped within a 560-kb chromosome region containing *S*-*RNase* genes. In the present study, no recombination between *S*-*RNase* and *PpSFBB* ^−*γ*^ was observed in 103 F1 plants from the Niitaka × 415-1 cross. Therefore, based on the results from our crossing experiments, the duplicated region that encompasses recombined SSR loci between the *S*-loci would contains all of the genes involved in CI between the *S*_*4*_- and *S*_*5*_-haplotypes.

Partial chromosomal duplication involving an *S*-locus has been reported in PPMs in petunia (Brewbaker and Natarajan 1960) and tobacco (Pandey 1965; Golz et al. 1999); these duplications were produced by X- or γ-ray irradiation of pollen. PPMs with an extra *S*-allele often had an additional small chromosome, called a centric fragment (Brewbaker and Natarajan 1960). More recently, the presence of an *S*-gene in a centric fragment has been detected by fluorescence in situ hybridization (FISH) analysis (Golz et al. 2001). On the other hand, PPMs without centric fragments have also been found (Pandey 1965; Golz et al. 1999); in these cases, the extra *S*-allele was either translocated to a non-homologous chromosome or inserted next to the original *S*-locus (Golz et al. 2001). Consequently, these three cases (i.e., existence as a centric fragment, translocation to a non-homologous chromosome, or linkage to the original *S*-locus) should be considered for the interpretation of our results.

If the duplicated segment identified in this study is present as a centric fragment or as part of a non-homologous chromosome, it would be inherited independently from LG17, and offspring inheriting the original *S*_*4*_- or *S*_*5*_-haplotype from 415-1 would occur in equal frequencies. For example, if 415-1 has a single copy of a duplicated *S*_*5*_-haplotype (designated *dS*_*5*_) unlinked to the normal *S*-locus, the expected segregation ratio of the pollen *S*-haplotype would be *S*_*4*_:*S*_*5*_:*S*_*4*_ *dS*_*5*_:*S*_*5*_ *dS*_*5*_ = 1:1:1:1 (Table 3). However, the probability of inheriting a duplicated *S*-haplotype is often less than that of a normal *S*-haplotype (Pandey 1967). Thus, if the probability of inheriting *dS*_*5*_ is represented as *x* (0 ≤ *x* ≤ 1, where 1 means transmission comparable to that of a non-duplicated allele), and the deletion of *dS*_*5*_-containing pollen occurs with probability (1–*x*), the segregation ratio of pollen *S*-haplotype would be *S*_*4*_:*S*_*5*_:*S*_*4*_ *dS*_*5*_:*S*_*5*_ *dS*_*5*_ = 1 + (1–*x*):1 + (1–*x*):*x*:*x*. Because *dS*_*5*_ and *S*_*5*_ cannot be distinguished electrophoretically, the segregation of pollen electrophoretic haplotypes under this scenario would be *S*_*4*_:*S*_*5*_:*S*_*4*_ *S*_*5*_ = (2–*x*):2:*x*. Because *x* ≤ 1, the frequency of offspring inheriting only an electrophoretic *S*_*4*_-haplotype from 415-1 will be more than half the frequency of those inheriting only an electrophoretic *S*_*5*_-haplotype.

**Table 3:** Comparison between observed and expected segregation of the electrophoretic *S*-phenotype under two hypotheses of *S*_*5*_-haplotype duplication

Parents and *S*-haplotypes	Number of seedlings	Hypothesized chromosomal location of duplicated *S*-haplotype^a^	Expected segregation of *S*-haplotype in pollen^b^	Expected segregation of *S*-haplotype in progeny^b^	Expected segregation of electrophoretic *S*-phenotype^b^	Observed segregation of electrophoretic *S*-phenotype
Niitaka (*S*_*3*_ *S*_*9*_) SI × 415-1 (*S*_*4*_ *S*_*5*_) PPM	103	*S*_*4*_|*S*_*5*_ *dS*_*5*_|−	*S*_*4*_:*S*_*5*_:*S*_*4*_ *dS*_*5*_:*S*_*5*_ *dS*_*5*_ = 1:1:1:1	*S*_*3*_ *S*_*4*_:*S*_*3*_ *S*_*5*_:*S*_*4*_ *S*_*9*_:*S*_*5*_ *S*_*9*_:*S*_*3*_ *S*_*5*_ *dS*_*5*_:*S*_*5*_ *dS*_*5*_ *S*_*9*_:*S*_*3*_ *S*_*4*_ *dS*_*5*_:*S*_*4*_ *dS*_*5*_ *S*_*9*_ = 1:1:1:1:1:1:1:1	*S*_*3*_ *S*_*4*_:*S*_*3*_ *S*_*5*_:*S*_*4*_ *S*_*9*_:*S*_*5*_ *S*_*9*_:*S*_*3*_ *S*_*4*_ *S*_*5*_:*S*_*4*_ *S*_*5*_ *S*_*9*_ = 1:2:1:2:1:1	*S*_*3*_ *S*_*4*_:*S*_*3*_ *S*_*5*_:*S*_*4*_ *S*_*9*_:*S*_*5*_ *S*_*9*_:*S*_*3*_ *S*_*4*_ *S*_*5*_:*S*_*4*_ *S*_*5*_ *S*_*9*_ = 2:40:0:47:7:7
		*S*_*4*_ *dS*_*5*_|*S*_*5*_	*S*_*4*_:*S*_*5*_:*S*_*4*_ *dS*_*5*_:*S*_*5*_ *dS*_*5*_ = 0:1:1:0	*S*_*3*_ *S*_*4*_:*S*_*3*_ *S*_*5*_:*S*_*4*_ *S*_*9*_:*S*_*5*_ *S*_*9*_:*S*_*3*_ *S*_*5*_ *dS*_*5*_:*S*_*5*_ *dS*_*5*_ *S*_*9*_:*S*_*3*_ *S*_*4*_ *dS*_*5*_:*S*_*4*_ *dS*_*5*_ *S*_*9*_ = 0:1:0:1:0:0:1:1	*S*_*3*_ *S*_*4*_:*S*_*3*_ *S*_*5*_:*S*_*4*_ *S*_*9*_:*S*_*5*_ *S*_*9*_:*S*_*3*_ *S*_*4*_ *S*_*5*_:*S*_*4*_ *S*_*5*_ *S*_*9*_ = 0:1:0:1:1:1	

^a^ *dS*_*5*_ Duplicated *S*_*5*_-haplotype, *S*_*4*_|*S*_*5*_ *dS*_*5*_|− duplicated *S*_*5*_-haplotype present as a centric fragment or translocated to a non-homologous chromosome, *S*_*4*_ *dS*_*5*_|*S*_*5*_ duplicated *S*_*5*_-haplotype tightly linked to *S*_*4*_-haplotype of homologous chromosome

^b^Expected segregation in the case that the probability of inheriting the duplicated *S*-haplotype is 1 (i.e., not reduced relative to that of a non-duplicated haplotype)

If the duplicated *S*_*5*_-haplotype is translocated or inserted very close to the original *S*_*4*_-haplotype (i.e., the chromosomes with *S*-haplotypes contain either *S*_*4*_ *dS*_*5*_ or *S*_*5*_), there would be little or no recombination between *dS*_*5*_ and the original *S*-locus, and the segregation ratio of pollen *S*-haplotypes would be *S*_*4*_:*S*_*5*_:*S*_*4*_ *dS*_*5*_:*S*_*5*_ *dS*_*5*_ = 0:1:1:0 (Table 3).

In our study, very few plants (<2 %) inherited the *S*_*4*_-haplotype alone from 415-1 (Table 3). Therefore, the chromosomal segment containing the duplicated *S*_*5*_-haplotype was usually inherited together with the *S*_*4*_ chromosome. Consequently, it is reasonable to infer that LG 17 of 415-1 is represented by one chromosome containing an *S*_*5*_-haplotype and a homologous chromosome containing both an *S*_*4*_-haplotype and a duplicate of the *S*_*5*_-haplotype (Fig. 2).

However, there remains a question as to why the frequency of plants from *S*_*4*_ *S*_*5*_ pollen (16 % of the frequency of progeny from *S*_*5*_ pollen) is so low, despite the prediction that *S*_*4*_ *S*_*5*_ pollen grains might be produced at the same frequency as *S*_*5*_ pollen. One possibility is that duplication of the *S*-haplotype or other adjacent genes might be detrimental to the production or growth of pollen. In trisomics of barley and tobacco, pollen grains with an extra chromosome were less frequent and smaller than normal grains, and were not fully mature at anthesis (Tsuchiya 1960; Niizeki and Saito 1988). Thus, reduced viability of heteroallelic pollen by segmental duplication could reduce the probability of inheriting the duplicated *S*_*5*_-haplotype. It is also possible that the duplicated *S*_*5*_-haplotype and the *S*_*4*_-haplotype are not very tightly linked (i.e., that recombination occurs at some frequency). Hence, at least three factors—the probability of deletion of the duplicated *S*-haplotype, the reduction of viability of heteroallelic pollen, and the possibility of recombination between *S*_*4*_ and *dS*_*5*_—are required to estimate the location of the duplicate *S*_*5*_-haplotype by segregation analysis of progeny from crosses involving 415-1. Therefore, our segregation data cannot be statistically compared with the basic models presented in Table 3, because one or more of these complicating factors affect the inheritance of the duplicated region. The two plants that inherited the *S*_*4*_-haplotype alone from 415-1 are assumed to be the product of “*S*_*4*_ pollen” that lost the duplicated *S*_*5*_ segment by deletion or unequal crossing-over.

### Variation among SI/SC systems in the Rosaceae

In the present study, a segmental duplication including the *S*-haplotype was found to induce pollen-part SC in Japanese pear. Pollen-part SC in the Pyrinae can also be caused by tetraploidization, as was reported for European pear (Crane and Lewis 1942), apple (Adachi et al. 2009), and Japanese pear (Tahira et al. 2010). Thus, acquisition of one or more extra *S*-haplotypes by either tetraploidization or segmental duplication can produce pollen-part SC in the Pyrinae, as is the case in the Solanaceae. Therefore, the mechanism of SI in the Pyrinae may be identical or closely related to that found in the Solanaceae (de Franceschi et al. 2012).

On the other hand, there are contradictory findings from two different tetraploid species of *Prunus* (Rosaceae), sour cherry (*Prunus cerasus* L.) and Chinese cherry (*Prunus pseudocerasus* Lindl.), carrying functional *S*-haplotypes. Sour cherry is not self-compatible (Hauck et al. 2006) whereas Chinese cherry is (Huang et al. 2008). These examples raise the question of whether the SI regulation mechanism is different in different species of *Prunus*, or whether the regulation mechanism is the same but leads to different responses (Tao and Iessoni 2010).

In a recent study of tetraploid Chinese cherry, Gu et al. (2013) showed that *S*-heteroallelic pollen with particular combinations of two different *S*-haplotypes, each containing a functional *SFB* gene, gave rise to SC whereas other combinations did not, and they concluded that the capability of the pollen to degrade *S*-RNase in the self style and to grow into the ovaries depends on the combination of *S*-haplotypes. Lewis (1943) had previously reported that heteroallelic pollen gave rise to SC in some cases but not in others, depending on the particular combination of *S*-haplotypes, in tetraploid *Oenothera organensis* Munz (Onagraceae). Under the CI hypothesis, *S*-heteroallelic pollen produced by a biallelic plant should be self-compatible whether one or more F-box proteins recognize and degrade self or non-self *S*-RNase. Therefore, the failure of breakdown of SI in *P. cerasus* might be the result of an incompatible combination of *S*-haplotypes and subsequent lack of *S*-RNase degradation, even though (as a general rule) CI occurs in heteroallelic pollen. Consequently, further investigations of PPMs with duplicated *S*-haplotypes or tetraploid PPMs will be required to determine whether all possible combinations of *S*-haplotypes in *S*-heteroallelic pollen give rise to CI in Japanese pear, as is seen for the *S*_*4*_ *S*_*5*_ pollen of 415-1.

## Conclusions

415-1 is the first diploid PPM with a duplicated *S*-haplotype to be identified in the Pyrinae. 415-1 can cross with normal diploid cultivars of any *S*-genotype, and an additional *S*-haplotype is transmitted to the progeny. More information about the chromosome status of the duplicated segment will be obtained by analyzing the inheritance of the duplicated *S*-allele. Our research group has started to analyze the phenotypes of different combinations of normal *S*-haplotypes and extra *S*-haplotype copies by using progeny of 415-1, which will enable the genetic analysis and selection of pollen-part SC by using the markers associated with the duplicated *S*-haplotype as indicators.

Based on the data reported here, the duplicated *S*_*5*_ chromosomal segment of 415-1 contains the genes necessary to cause CI with the *S*_*4*_-haplotype. In a case such as this which involves a relatively small duplicated region, the effect of genetic factors outside of the *S*-haplotype itself (e.g., modifying factors) would be much more limited than in the case of whole-genome duplication, as is found in tetraploid PPMs. Therefore, materials derived from 415-1 will be valuable for elucidating the mechanisms of regulation and function of SI, and especially that of CI caused by a duplicated *S*-haplotype. We plan to search for genes involved in SI and SC in the duplicated region by using genome information from apple (Velasco et al. 2010). Further studies of 415-1 and its offspring will help to elucidate the self/non-self recognition mechanism between pollen and pistil in pear and its relatives.

## References

[CR1] Adachi Y, Komori S, Hoshikawa Y, Yanaka N, Abe K, Bessho H, Watanabe M, Suzuki A (2009). Characteristics of fruiting and pollen tube growth of apple autotetraploid cultivars showing self-compatibility. J Jpn Soc Hortic Sci.

[CR2] Bošković RI, Wolfram B, Tobutt KR, Cerović R, Sonneveld T (2006). Inheritance and interactions of incompatibility alleles in the tetraploid sour cherry. Theor Appl Genet.

[CR3] Brewbaker JL, Natarajan AT (1960). Centric fragments and pollen part mutation of incompatibility alleles in Petunia. Genetics.

[CR4] Celton JM, Tustin DS, Chagné D, Gardiner SE (2009). Construction of a dense genetic linkage map for apple rootstocks using SSRs developed from *Malus* ESTs and *Pyrus* genomic sequences. Tree Genet Genomes.

[CR5] Crane MB, Lewis D (1942). Genetical studies in pears. III. Incompatibility and sterility. J Genetics.

[CR6] de Franceschi P, Pierantoni L, Dondini L, Grandi M, Sanzol J, Sansavini S (2011). Cloning and mapping S-locus F-box genes in European pear (*Pyrus communis* L.). Tree Genet Genomes.

[CR7] de Franceschi P, Dondini L, Sanzol J (2012). Molecular bases and evolutionary dynamics of self-incompatibility in the Pyrinae (Rosaceae). J Exp Bot.

[CR8] de Nettancourt D (2001). Incompatibility and incongruity in wild and cultivated plants.

[CR9] de Nettancourt D, Dijkhuis P, van Gastel AJG, Broertjes C (1971). The combined use of leaf irradiation and of the adventitious bud technique for inducing and detecting polyploidy, marker mutations and self-compatibility in clonal populations of *Nicotiana alata* Link and Otto. Euphytica.

[CR10] Entani T, Iwano M, Shiba H, Che FS, Isogai A, Takayama S (2003). Comparative analysis of the self-incompatibility (*S*-) locus region of *Prunus mume*: identification of a pollen-expressed F-box gene with allelic diversity. Genes Cells.

[CR11] Gianfranceschi L, Seglias N, Tarchini R, Komjanc M, Gessler C (1998). Simple sequence repeats for the genetic analysis of apple. Theor Appl Genet.

[CR12] Golz JF, Su V, Clarke AE, Newbigin E (1999). A molecular description of mutations the pollen component of the *Nicotiana alata S* locus. Genetics.

[CR13] Golz JF, Su V, Oh HY, Kusaba M, Newbigin E (2001). Genetic analysis of *Nicotiana* pollen-part mutants is consistent with the presence of an *S*-ribonuclease inhibitor at the *S* locus. Proc Natl Acad Sci USA.

[CR14] Gu C, Liu QZ, Yang YN, Zhang SJ, Khan MA, Wu J, Zhang SL (2013) Inheritance of hetero-diploid pollen *S*-haplotype in self-compatible tetraploid Chinese cherry (*Prunus pseudocerasus* Lindl). PLoS One 8. doi:10.1371/journal.pone.0061219 10.1371/journal.pone.0061219 PMC3626605 23596519

[CR15] Hauck NR, Yamane H, Tao R, Iezzoni AF (2006). Accumulation of non-functional *S*-haplotypes results in the breakdown of gametophytic self-incompatibility in tetraploid Prunus. Genetics.

[CR16] Huang SX, Wu HQ, Li YR, Wu J, Zhang SJ, Heng W, Zhang SL (2008). Competitive interaction between two functional *S*-haplotypes confer self-compatibility on tetraploid Chinese cherry (*Prunus pseudocerasus* Lindl. cv. Nanjing Chuisi). Plant Cell Rep.

[CR17] Kakui H, Tsuzuki T, Koba T, Sassa H (2007). Polymorphism of *SFBB*^−*γ*^ and its use for *S* genotyping in Japanese pear (*Pyrus pyrifolia*). Plant Cell Rep.

[CR18] Kakui H, Kato M, Ushijima K, Kitaguchi M, Kato S, Sassa H (2011). Sequence divergence and loss-of-function phenotypes of *S locus F*-*box brothers* (*SFBB*) genes are consistent with non-self recognition by multiple pollen determinants in self-incompatibility of Japanese pear (*Pyrus pyrifolia*). Plant J.

[CR19] Kosambi DD (1944). The estimation of map distances from recombination values. Ann Hum Genet.

[CR20] Kubo K, Entani T, Takara A, Wang N, Fields AM, Hua Z, Toyoda M, Kawashima S, Ando T, Isogai A, Kao T, Takayama S (2010). Collaborative non-self recognition system in S-RNase-based self-incompatibility. Science.

[CR21] Lai Z, Ma WS, Han B, Liang LZ, Zhang YS, Hong GF, Xue YB (2002). An F-box gene linked to the self-incompatibility (*S*) locus of *Antirrhinum* is expressed specifically in pollen and tapetum. Plant Mol Biol.

[CR22] Lewis D (1943). Physiology of incompatibility in plants III. Autopolyploids. J Genet.

[CR23] Lewis D (1952). Serological reactions of pollen incompatibility substances. Proc R Soc Lond B Biol Sci.

[CR24] Lewis D, Modlibowska I (1942). Genetical studies in pears. IV. Pollen-tube growth and incompatibility. J Genet.

[CR25] Liebhard R, Gianfranceschi L, Koller B, Ryder CD, Tarchini R, van de Weg E, Gessler C (2002). Development and characterisation of 140 new microsatellites in apple (*Malus* × *domestica* Borkh.). Mol Breed.

[CR26] McClure BA, Haring V, Ebert PR, Anderson MA, Simpson RJ, Sakiyama F, Clarke AE (1989). Style self-incompatibility gene products of *Nicotiana alata* are ribonucleases. Nature.

[CR27] Minamikawa M, Kakui H, Wang S, Kotoda N, Kikuchi S, Koba T, Sassa H (2010). Apple *S* locus region represents a large cluster of related, polymorphic and pollen-specific F-box genes. Plant Mol Biol.

[CR28] Niizeki M, Saito K (1988). Increasing the transmission rate of the extra chromosome in a trisomic *Nicotiana sylvestris* line by modifying the means of pollination. Theor Appl Genet.

[CR29] Nishitani C, Terakami S, Sawamura Y, Takada N, Yamamoto T (2009). Development of novel EST-SSR markers derived from Japanese pear (*Pyrus pyrifolia*). Breed Sci.

[CR30] Norioka N, Norioka S, Ohnishi Y, Ishimizu T, Oneyama C, Nakanishi T, Sakiyama T (1996). Molecular cloning and nucleotide sequence of cDNAs encoding *S*-allele specific stylar RNases in a self-incompatible cultivar and its mutant of Japanese pear, *Pyrus pyrifolia* Nakai. J Biochem.

[CR31] Okada K, Tonaka N, Moriya Y, Norioka N, Sawamura Y, Matsumoto T, Nakanishi T, Takasaki-Yasuda T (2008). Deletion of a 236 kb region around *S*_4_-*RNase* in a stylar-part mutant *S*_4_^*sm*^-haplotype of Japanese pear. Plant Mol Biol.

[CR32] Okada K, Tonaka N, Taguchi T, Ichikawa T, Sawamura Y, Nakanishi T, Takasaki-Yasuda T (2011). Related polymorphic F-box protein genes between haplotypes clustering in the BAC contig sequences around the *S*-*RNase* of Japanese pear. J Exp Bot.

[CR33] Pandey KK (1965). Centric chromosome fragments and pollen-part mutation of the incompatibility gene in *Nicotiana alata*. Nature.

[CR34] Pandey KK (1967). Elements of *S*-gene complex. II. Mutation and complementation at the SI locus in *Nicotiana alata*. Heredity.

[CR35] Royo J, Kunz C, Kowyama Y, Anderson M, Clarke AE, Newbigin E (1994). Loss of a histidine residue at the active site of *S*-locus ribonuclease is associated with self-compatibility in *Lycopersicon peruvianum*. Proc Natl Acad Sci USA.

[CR36] Saito T, Sato T, Sawamura Y, Shoda M, Takasaki-Yasuda T, Kotobuki K (2012). Dual recognition of *S*_*1*_ and *S*_*4*_ pistils by *S*_*4*_^*sm*^ pollen in self-incompatibility of Japanese pear (*Pyrus pyrifolia* Nakai). Tree Genet Genomes.

[CR37] Sassa H, Nishio T, Kowyama Y, Hirano H, Koba T, Ikehashi H (1996). Self-incompatibility (*S*) alleles of the Rosaceae encode members of a distinct class of the T2/S ribonuclease superfamily. Mol Gen Genet.

[CR38] Sassa H, Hirano H, Nishino T, Koba T (1997). Style-specific self-compatible mutation caused by deletion of the S-RNase gene in Japanese pear (*Pyrus serotina*). Plant J.

[CR39] Sassa H, Kakui H, Miyamoto M, Suzuki Y, Hanada T, Ushijima K, Kusaba M, Hirano H, Koba T (2007). *S locus F*-*box brothers*: multiple and pollen-specific F-box genes with *S* haplotype-specific polymorphisms in apple and Japanese pear. Genetics.

[CR40] Sawamura Y, Mase N, Takada N, Sato A, Nishitani C, Kazuyuki A, Masuda T, Yamamoto T, Saito T, Kotobuki K (2013). A self-compatible pollen-part mutant of Japanese pear produced by crossing ‘Kosui’ with pollen from gamma-irradiated ‘Kosui’. J Jpn Soc Hortic Sci.

[CR41] Schnable PS, Ware D, Fulton RS (2009). The B73 maize genome: complexity, diversity, and dynamics. Science.

[CR42] Sijacic P, Wang X, Skirpan AL, Wang Y, Dowd PE, McCubbin AG, Huang S, Kao TH (2004). Identification of the pollen determinant of S-RNase-mediated self-incompatibility. Nature.

[CR43] Silfverberg-Dilworth E, Matasci CL, Van de Weg WE, Van Kaauwen MPW, Walser M, Kodde LP, Soglio V, Gianfranceschi L, Durel CE, Costa F, Yamamoto T, Koller B, Gessler C, Patocchi A (2006). Microsatellite markers spanning the apple (*Malus* × *domestica* Borkh.) genome. Tree Genet Genomes.

[CR44] Sonneveld T, Tobutt KR, Vaughan SP, Robbins TP (2005). Loss of pollen-*S* function in two self-compatible selections of *Prunus avium* is associated with deletion/mutation of an *S* haplotype-specific F-box gene. Plant Cell.

[CR45] Tahira H, Yonemura Y, Takeuchi R, Yamashita M, Endo T, Morimoto T, Otsu S (2010) Method for creating polyploid of plant of genus *Pyrus*, and polyploid of genus *Pyrus*. Japan Patent, 2010-104273A (pending)

[CR46] Takasaki T, Okada K, Castillo C, Moriya Y, Saito T, Sawamura Y, Norioka N, Norioka S, Nakanishi T (2004). Sequence of the *S*_9_-RNase cDNA and PCR-RFLP system for discriminating *S*_1_- to *S*_9_-allele in Japanese pear. Euphytica.

[CR47] Tao R, Iessoni AF (2010). The S-RNase-based gametophtic self-incompatibility system in *Prunus* exhibit distinct genetic and molecular features. Sci Hortic.

[CR48] Terakami S, Kimura T, Nishitani C, Sawamura Y, Saito T, Hirabayashi T, Yamamoto T (2009). Genetic linkage map of the Japanese pear ‘Housui’ identifying three homozygous genomic regions. J Jpn Soc Hortic Sci.

[CR49] Tsuchiya T (1960). Cytogenetic studies of trisomics in barley. Jpn J Bot.

[CR50] Ushijima K, Sassa H, Dandekar AM, Gradziel TM, Tao R, Hirano H (2003). Structural and transcriptional analysis of the self-incompatibility locus of almond: identification of a pollen-expressed F-box gene with haplotype-specific polymorphism. Plant Cell.

[CR51] Ushijima K, Yamane H, Watari A, Kakehi E, Ikeda K, Hauck N, Iezzoni A, Tao R (2004). The *S* haplotype-specific F-box gene, *SFB*, is defective in self-compatible haplotypes of *Prunus avium* and *P. mume*. Plant J.

[CR52] Velasco R, Zharkikh A, Affourtit J (2010). The genome of the domesticated apple (*Malus* × *domestica* Borkh.). Nat Genet.

[CR53] Xue YB, Carpenter R, Dickinson HG, Coen ES (1996). Origin of allelic diversity in antirrhinum *S* locus RNases. Plant Cell.

[CR54] Yamamoto T, Kimura T, Shoda M, Ban Y, Hayashi T, Matsuta N (2002). Development of microsatellite markers in Japanese pear (*Pyrus pyrifolia* Nakai). Mol Ecol Notes.

[CR55] Yamamoto T, Kimura T, Shoda M, Imai T, Saito T, Sawamura Y, Kotobuki K, Hayashi T, Matsuta N (2002). Genetic linkage maps constructed by using an interspecific cross between Japanese and European pears. Theor Appl Genet.

[CR56] Yamamoto T, Kimura T, Terakami S, Nishitani C, Sawamura Y, Saito T, Kotobuki K, Hayashi T (2007). Integrated reference genetic linkage maps of pear based on SSR and AFLP markers. Breed Sci.

[CR57] Yamane H, Ikeda K, Ushijima K, Sassa H, Tao R (2003). Self-incompatibility (*S*) locus region of the mutated *S*^6^-haplotype of sour cherry (*Prunus cerasus*) contains a functional pollen *S* allele and a non-functional pistil *S* allele. J Exp Bot.

